# NLRP3 inflammasome in peripheral blood monocytes as a risk factor for early -onset preeclampsia

**DOI:** 10.1186/s12884-023-05606-5

**Published:** 2023-05-24

**Authors:** Hong Yang, Mo Ai, Yanqiu Guo, Bingfen Li, Cong Liu, Dongying Qu

**Affiliations:** Department of Gynaecology and Obstetrics, The General Hospital of Northern Theater Command, No. 83 Wenhua Road, Shenhe District, Shenyang City, China

**Keywords:** Early-onset preeclampsia, NLRP3 inflammasome, Real-time PCR, Logistic regression

## Abstract

**Introduction:**

As a leading cause of pregnancy and fetal mortality, pre-eclampsia impacts about 5–8% of pregnancies globally. To date, few studies have focused on the role played by (NOD)-like receptors protein 3 (NLRP3) in peripheral blood in early-onset pre-eclampsia (PE). In this study, we investigated whether NLRP3 expression in monocytes before 20 weeks of gestation was associated with an increased risk of early-onset PE.

**Methodology:**

During the study period from 2019 to 2021, women with singleton pregnancies were enrolled in this prospective study at the General Hospital of Northern Theater Command. A generalized additive model (GAM) and logistic regression models were applied to determine any association between NLRP3 and the risk of early-onset PE.

**Results:**

In total, 571 and 48 subjects were included in the control and pre-eclampsia groups, respectively. The GAM and logistic regression models showed that NLRP3 was a significant factor for PE occurrence. The area under the curve, accuracy, specificity, sensitivity, positive likelihood ratio, negative likelihood ratio, and diagnostic odds ratio were 0.86, 0.82, 0.95, 0.72, 15.17, 0.29, and 52.0, respectively.

**Conclusion:**

The monitoring for NLRP3 in peripheral blood may be a potential, prospectively identifying risk factor for preeclampsia.

## Introduction

Preeclampsia (PE) is a leading cause of pregnancy and fetal mortality that affects about 5–8% of pregnancies worldwide [1, 2]. The early-onset PE generally occurs after 20 weeks and before 34 weeks of gestation, but the clinical manifestation and mechanism of PE are complicated. Preeclampsia is considered a 2-stage disorder; defective trophoblast invasion and spiral artery remodeling failure are postulated as the primary step responsible for preeclampsia pathogenesis [3]. The pathophysiology of PE was strongly connected with shallow invasion [4]. Previous studies have shown that the placenta plays a crucial role in the mechanism of PE, and the disease symptoms disappear rapidly after delivery [5]. The clinical features of PE reflect widespread systemic inflammation [6]. A series of studies have found that the inflammatory cytokines increase because of trophoblast cells during the first trimester of pregnancy, indicating that inflammatory cytokines play an important role in the development of PE [7–15].

Previous studies have shown that nucleotide-binding oligomerization domain (NOD)-like receptors (NLRs) are associated with the expression of several inflammatory factors, such as IL-1β. Of all the NLRs, NLRP3 is most extensively studied [14]. Once activated, NLRP3 comprises a complex that can process pro-IL-1β into IL-1β [16, 17]. The mature form of IL-1β produces an inflammatory response in the endothelium of pregnant women and influences maternal spiral artery remodeling. Several recent studies have found that the NLRP3 inflammasome plays an important role in the mechanism of PE [18, 19]. One study reported that the level of NLRP3 in the placental tissue of pregnant women with PE was higher than that in healthy controls [20]. The components of NLRP3, including ASC and caspase-1, can be expressed in immune cells and human trophoblast cells. In summary, NLRP3 activation and the consequent inflammatory reaction may play a key role in the occurrence of early-onset PE. However, few studies have focused on the role played by NLRP3 in peripheral blood in early-onset PE.

In the present study, we examined the expression of NLRP3 in peripheral blood monocytes before 20 weeks of gestation, and we analyzed the association between NLRP3 and the risk of PE. Indeed, the overall aim of this study was to assess whether NLRP3 expression in monocytes is related to an increased risk of early-onset PE.

## Materials and methods

### Subjects

**Fig. 1:**
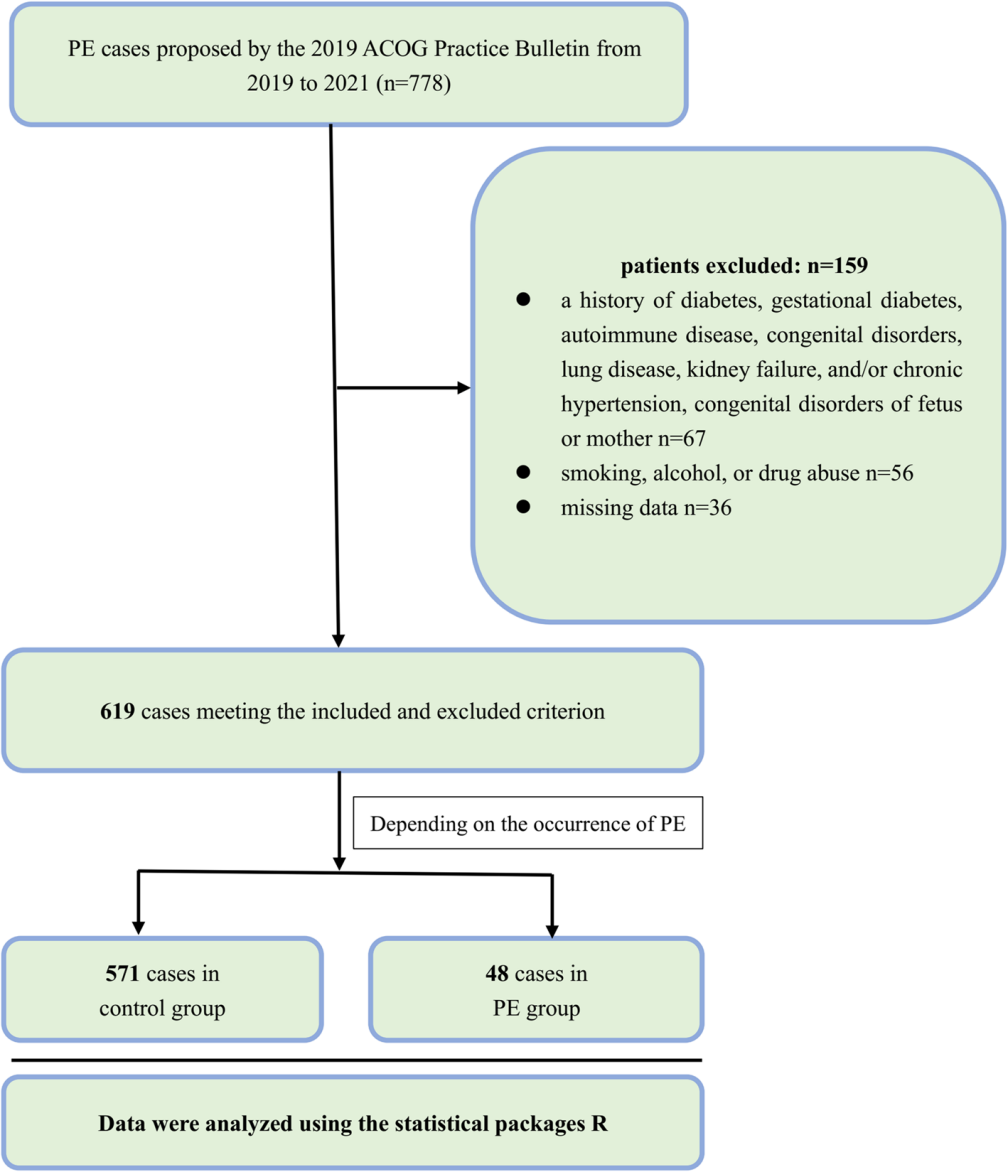
The flowchart of inclusion and exclusion criteria

Women with singleton pregnancies attending for prenatal screenings were enrolled in this prospective study during 2019–2021 at the General Hospital of Northern Theater Command. The diagnostic criteria proposed by the 2019 ACOG Practice Bulletin were used to diagnose PE, which was defined as gestational hypertension (≥ 140/90 mmHg) and proteinuria (urine protein ≥ 300 mg/24 h) after 20 weeks and before 34 weeks of gestation. The inclusion criteria included a one-fetus pregnancy and being aged 18–40 years, body mass index (BMI) = 18–30 kg/m^2^. The exclusion criteria were a history of diabetes, gestational diabetes, autoimmune disease, congenital disorders, lung disease, kidney failure, and/or chronic hypertension, congenital disorders of fetus or mother, as well as smoking, alcohol, or drug abuse as suggested by other studies (Fig. 1). Clinical data, including age, body mass index, parity, baby weight, smoking status, and family history of PE, were collected. These pregnant women were followed from the first trimester of pregnancy (10–14 weeks) to delivery. This study was approved by the Ethics Committee of the General Hospital of Northern Theater Command (NO.2019015), and all participants signed an informed consent form. The reporting of this study conforms to STROBE guidelines [21].

### Monocyte isolation and preparation

At 20 weeks of gestation, 5 mL of peripheral blood was collected from each participant. All peripheral blood samples were processed within 2 h of collection. The density gradient centrifugation method was used to isolate human peripheral blood monocytes, which were incubated at 37 °C with 5% CO_2_ for 2 h.

### RNA isolation and quantitative real-time RT-PCR

To analyze the expression of NLRP3 in all subjects, real-time PCR was performed. The total RNA of blood monocytes was extracted using Trizol (Life Technologies, 15,596–026; Invitrogen, USA) and stored at − 80 °C. A High-Capacity cDNA Archive Kit (Applied Biosystems) was used to synthesize cDNA. TaqManVR probes (Applied Biosystems, Hs00918082_m1) were used to determine the RNA expression of NLRP3. The expression ratios of mRNA were identified using the ΔΔCt (threshold cycle) method for relative quantification. Fold changes were calculated using GADPH as an endogenous control.

### Statistical analysis

Measurement data, including age, body mass index (BMI), baby weight, white blood cell count, and relative NLRP3 mRNA, are expressed as means ± SD. Enumerated data, including parity, smoking status, and family history of PE, are expressed as percentages. T-tests or χ2 tests were used to analyze the differences between groups. The relationship of NLRP3 with the risk of PE was identified using a generalized additive model and logistic regression models. The results were reported as odds ratios (ORs), 95% confidence intervals, and *p* values. Receiver operating characteristic curve (ROC) analysis and plotted decision curves were used to evaluate the reliability of the models for assessing the risk of PE. *P* values < 0.05 were considered statistically significant. Data were analyzed using statistical packages in R (The R Foundation; http://www.r-project.org; version 3.4.3).

## Results

### Clinical characteristics of patients

We enrolled 619 pregnant women in this study. Depending on the occurrence of PE, 571 and 48 pregnant women were included in the control and PE groups, respectively. There were no significant differences in terms of age, parity, smoking status, family history of PE, and white blood cell count (Table 1).

**Table 1:** Clinical summary of patients’ data

Characteristics	Control group (*N* = 571)	Preeclampsia group (*N* = 48)	*P*
**Age(year)**	27.04 ± 4.26	28.06 ± 4.04	0.11
**Body mass index(kg/m**^**2**^**)**	22.60 ± 1.52	23.36 ± 2.04	0.001
**Parity**			0.53
*Primipara (n, %)*	265(46.4%)	20(41.7%)	
*Multipara (n, %)*	306(53.6%)	28(58.3%)	
**Baby weight(kg)**	3.46 ± 0.51	2.88 ± 0.66	< 0.001
**Smoking (n, %)**	61(10.7%)	6(12.5%)	0.69
**Family history of PE (n, %)**	56(9.8%)	4(8.3%)	0.74
**White blood cell(10^9/L)**	8.61 ± 1.61	8.82 ± 1.50	0.38
**Menarche (year)**	13.21 ± 2.34	13.32 ± 3.15	0.76
**Previous abortions (n, %)**	51(8.9%)	5(10.4%)	0.73
**Gestational age at delivery (week)**	39.23 ± 2.15	39.45 ± 1.56	0.49
**Ethnicity**			0.66
*Asian (n, %)*	553(96.8%)	46(95.8%)	
*others (n, %)*	18(3.2%)	2(4.2%)	
**Relative NLRP3 mRNA**	1.16 ± 0.29	2.21 ± 0.76	< 0.001

In the PE group, the average BMI was 23.36 ± 2.04 kg/m^2^, which was higher than that in the control group (22.60 ± 1.52 kg/m^2^; *p* = 0.003). In the PE group, the average baby weight was 2.88 ± 0.66 kg, which was lower than that in control group (3.46 ± 0.51 kg; *p* < 0.001). In the PE group, the average relative NLRP3 mRNA level was 2.21 ± 0.76, which was higher than that in control group (1.16 ± 0.29; *p* < 0.001).

### Linear relationship between NLRP3 and PE

**Fig. 2:**
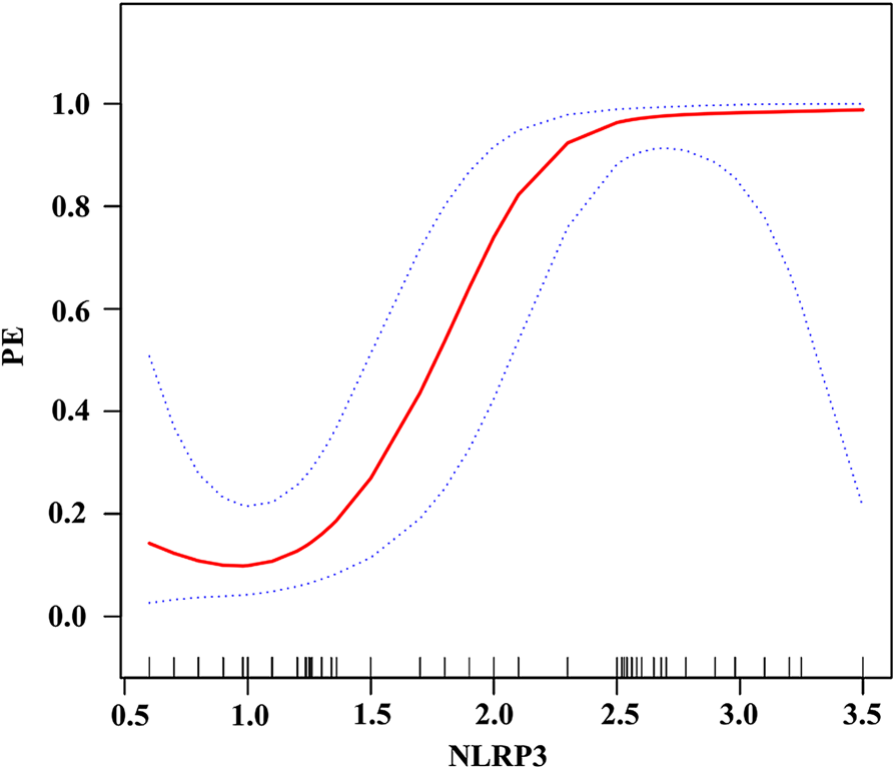
Linear relationship between NLRP3 and pre-eclampsia. The red and black points represent the fitting spline and 95% confidence intervals, respectively

The smoothing spline was used to analyze the relationship between NLRP3 and PE after adjusting for parity, baby weight, age, smoking status, BMI, family history of PE, and white blood cell count. As shown in Fig. 2, a linear relationship existed between NLRP3 and PE (β = 3.2, *p* < 0.0001; the red and black points show the fitting spline and the 95% confidence intervals, respectively). NLRP3 varied from 1.1 to 2.5 and was significantly correlated with the occurrence of PE (OR = 54.8, *p* < 0.001; Table 2).

**Table 2:** Threshold effect analysis of NLRP3 on preeclampsia using piece-wise linear regression

Inflection point of NLRP3	OR	95%CI	*P*
≤ 1.1	0.01	0.002,1.9	0.088
1.1–2.5	54.8	6.2,164.0	< 0.001
** > **2.5	0.02	0.001,21.3	0.323

### Relationship between NLRP3 and PE in different models

Multivariate logistic regression was used to analyze the association between NLRP3 and PE (Table 3). In the crude model and the adjusted I (adjusting for age) and II (adjusting for age, BMI, parity, baby weight, smoking status, family history of PE, and white blood cell count) models, NLRP3 was a significant factor in PE occurrence (Crude: OR = 16.3, *p* < 0.001; Adjusted I: OR = 17.6, *p* < 0.001; Adjusted II: OR = 22.0, *p* < 0.001).

**Table 3:** Individual effect of NLRP3 on preeclampsia

Parameters	Non-adjusted		Adjust model I		Adjust model I	
	OR (95%CI)	*P* value	OR (95%CI)	*P* value	OR (95%CI)	*P* value
**Continuous NLRP3**	16.3 (7.2, 37.2)	< 0.001	17.6 (7.1, 44.0)	< 0.001	22.0 (7.5, 64.3)	< 0.001

Model I: adjusting for age

Model II: adjusting for age, BMI, parity, baby weight, smoking, family history of PE and white blood cell

### Model assessment

**Fig. 3:**
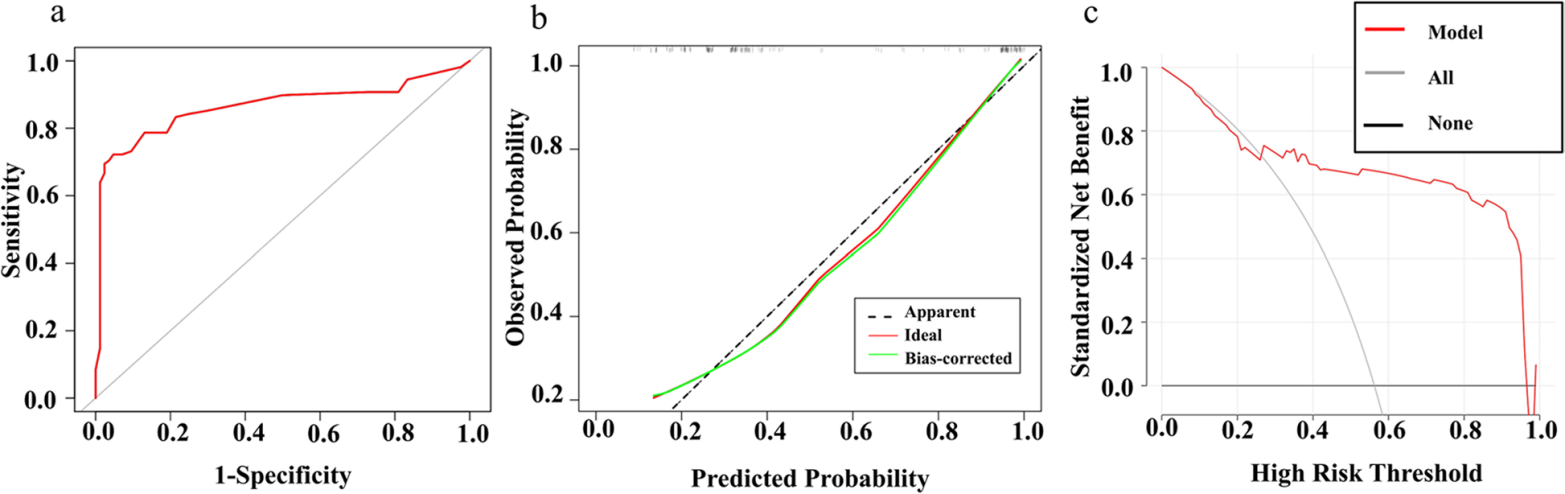
Assessment of the model used to predict the risk of pre-eclampsia (PE). The area under the curve of the model used to predict the risk of PE was 0.86 (**a**). Calibration curves showed that the predicted outcome fitted well to the observed outcome (**b**). According to the decision curve, using NLRP3 to predict PE risk was more beneficial than the “All or None” scheme if the threshold probability was > 25% and < 96% (**c**)

ROC analysis was used to assess the reliability of the models for predicting the risk of PE (Fig. 3a). The area under the curve, accuracy, specificity, sensitivity, positive likelihood ratio, negative likelihood ratio, and diagnostic odds ratio were 0.86, 0.82, 0.95, 0.72, 15.17, 0.29, and 52.0, respectively (Table 4). Calibration curves showed that the predicted outcome fitted well to the observed outcome (Fig. 3b). Decision curves showed that using NLRP3 to predict PE risk was more beneficial than using the “All or None” scheme if the threshold for probability was > 25% and < 96% (Fig. 3c), which suggests that NLRP3 data performs relatively well when clinically applied for the prediction of PE.

**Table 4:** ROC analysis results

Items	AUC	Accuracy	Specificity	Sensitivity	PLR	NLR	DOR
Model	0.86	0.82	0.95	0.72	15.17	0.29	52.0

*AUC* Area Under the Curve

*PLR* positive likelihood ratio

*NLR* negative likelihood ratio

*DOR* Diagnostic Odds Ratio

## Discussion

PE is a common obstetric complication that endangers the health of mothers and infants. However, there is no consensus on the relationship between NLRP3 in monocytes and early-onset PE. Our study focused on the expression of NLRP3 in monocytes before 20 weeks of pregnancy and evaluated the relationship between NLRP3 and PE. NLRP3 was found to be an independent risk factor for the occurrence of PE. Furthermore, ROC analysis and decision curves showed that NLRP3 data performed relatively reliably when applied clinically.

It is believed that damage to vascular endothelial cells, ischemia and hypoxia of the placenta, and shallow placental implantation comprise the physiological basis of PE [22–24]. Studies related to the placenta, cell nourishment, and ischemia have shown that the key mechanism of PE is the lack of invasion of the spiral arteries in the early stages of pregnancy, which causes some abnormal secretion of cytotoxic factors [9, 25]. Hypertension, proteinuria, and edema due to PE are all caused by cytotoxic factors [8, 26]. In pregnant women with PE, the activation of vascular endothelial cells caused by inflammation and placental abnormalities can lead to changes in the levels of various peripheral blood markers, some of which are altered earlier than the appearance of clinical symptoms [6, 27–29]. Oxidative stress in the placenta induces the release of placental factors into the maternal blood flow that further triggers endothelial dysfunction and enhanced vascular permeability. Ölmez, F. et al. found that maternal serum AQP9 concentrations were significantly increased in early-onset preeclampsia patients than healthy normotensive pregnant patients and suggested that AQP9 might be a crucial biomarker of the inflammatory process in early-onset preeclampsia [30]. Previous studies showed that the circulating maternal placental growth factor tests, in combination with uterine artery Doppler waveform assessments in the second trimester, may indicate the likely underlying type of placental pathology mediating severe adverse perinatal events. This approach has the potential to test disease-specific therapeutic strategies to improve clinical outcomes [31, 32]. Therefore, the use of a variety of peripheral blood markers for predicting the onset of eclampsia has clinical significance for the timely screening of PE.

A new term placental inflammation has come to practice in recent years. Previous studies showed that placental inflammation played an important role in the occurrence of PE. Beksac et al. found that low-dose low-molecular-weight heparin prophylaxis is useful to prevent metabolic and immunological disorders causing placental inflammation, which is the most likely pathophysiological mechanism contributing to PE [33]. The NLRP3 inflammasome is composed of NLRP3, ASC, and caspase-1. After its activation by a variety of substances, the NLRP3 inflammasome processes pro-IL-1β to IL-1β, which is released from the cell. IL-1β plays crucial roles in inflammatory reactions. Lee et al. found that pharmacological and genetic inhibition of TBK1 in trophoblasts ameliorated LPS-induced NLRP3 inflammasome activation, placental inflammation, and subsequent interleukin (IL)-1 production [34]. Previous studies have shown that the NLRP3 inflammasome can be expressed in the first trimester by trophoblast cells [35]. Compared with healthy controls, both placental and circulating IL-1β levels are increased in women with PE [36, 37]. Such women also show increased expression of the NLRP3 inflammasome and IL-1β mRNA in circulating neutrophils [38].

Hypertension is a characteristic of PE and the renin–angiotensin system is known to play an essential role in its mechanism. Ang II-induced hypertension in mice can be attenuated by inhibition of NLRP3 inflammasome activation [39, 40]. Moreover, salt-induced hypertension in rats occurs partly because of NLRP3 inflammasome activation [41]. It has also been demonstrated that the absence of ASC, a component of the NLRP3 inflammasome, reduces hypoxia-induced hypertension [42]. These findings indicate that the NLRP3 inflammasome has an important function in hypertension in pregnant women. Ozeki et al. demonstrated that S100A9 acts as a danger signal to activate the NLRP3 inflammasome in the placenta, associating with hypertension during pregnancy [43].

An accumulation of evidence now suggests that the NLRP3 inflammasome plays a pivotal role in the mechanism of inflammatory complications during pregnancy. Zeng et al. found that the activation of NLRP3 inflammasome in the uterus is responsible for the excessive inflammation at the maternal–fetal interface during PE [44]. The NLRP3 inflammasome processes an increased “danger” of cytokines after the NLRP3 is activated. Thus, it is essential to identify new targets for the treatment of PE after understanding the mechanism of the NLRP3 inflammasome in regulating pregnancy complications. Negi et al. reported that allopurinol could be a candidate medication to prevent PE by inhibit trophoblast secretion of inflammasome [45]. Moreover, Park et al. found that antioxidants could inhibit the expression of NLRP3 protein in trophoblast cells [37]. They might represent suitable therapeutic options for the treatment of PE. Therefore, how to inhibit activated inflammasomes could be an important target when preventing adverse pregnancy outcomes.

As far as I know, this is the first study to the current time that has assessed NLRP3 in the monocytes in patients with early-onset preeclampsia and compared them with normotensive pregnant women. Further studies are required in which NLRP3 in the monocytes and clinical outcomes are confirmed by postnatal histopathological examination.

## Conclusion

Overall, clinicians should pay more attention to expression of NLRP3 in the monocytes of pregnant women because it could have clinical significance in terms of avoiding adverse outcomes in pregnancy. We note that the bigger sample size would improve the reliability of study. Consequently, validation of the prognostication tool is still required.

## Data Availability

The data that support the findings of this study are available from the corresponding author upon reasonable request.

## Abbreviations

GAM: Generalized additive model
PE: Preeclampsia
NLRs: (NOD)-like receptors
NLRP3: (NOD)-like receptors protein 3
PCR: Polymerase Chain Reaction
BMI: Body mass index
ORs: Odds ratios
ROC: Receiver operating characteristic curve
